# Cognitive impairment in first-episode mania: a systematic review of the evidence in the acute and remission phases of the illness

**DOI:** 10.1186/s40345-015-0024-2

**Published:** 2015-04-25

**Authors:** Rothanthi Daglas, Murat Yücel, Sue Cotton, Kelly Allott, Sarah Hetrick, Michael Berk

**Affiliations:** Orygen, The National Centre of Excellence in Youth Mental Health, 35 Poplar Road, Parkville, VIC 3052 Australia; Centre for Youth Mental Health, The University of Melbourne, 35 Poplar Road, Parkville, VIC 3052 Australia; Monash Clinical and Imaging Neuroscience (MCIN), School of Psychological Sciences and Monash Biomedical Imaging Facility, Monash University, 770 Blackburn Rd, Clayton, VIC 3168 Australia; IMPACT Strategic Research Centre, School of Medicine, Deakin University, 288-299 Ryrie Street, PO Box 281, Geelong, VIC 3220 Australia; Barwon Health and the Geelong Clinic, Swanston Centre, 288-299 Ryrie Street, P O Box 281, Geelong, VIC 3220 Australia; Florey Institute for Neuroscience and Mental Health, Kenneth Myer Building, Royal Parade, Parkville, VIC 3220 Australia

**Keywords:** Mania, Cognition, Bipolar disorder, Depression, First episode, Early intervention

## Abstract

There is evidence of cognitive impairment that persists in the remission phase of bipolar disorder; however, the extent of the deficits that occur from the first onset of the disorder remains unclear. This is the first systematic review on cognitive functioning in the early stages of bipolar I disorder. The aim of the study was to identify the patterns and degree of cognitive impairment that exists from first-episode mania. Three electronic databases (MEDLINE, PsycINFO and PubMed) were systematically searched for studies published from January 1980 to June 2014. Eligible studies were separated into two groups: acute and remission. The Newcastle-Ottawa quality assessment scale was utilised to measure the quality of the included studies. A total of seven studies (three acute and four remission), including 230 first-episode mania and 345 healthy control participants, were eligible for the review. The studies in the acute phase only examined aspects of executive functioning, with impairments identified in cognitive flexibility, though not in response inhibition and verbal fluency relative to healthy controls. The most consistent finding during the remission phase was a deficit in working memory, whereas in the other domains, the findings were equivocal. Non-verbal memory and verbal fluency were not impacted in remission from first-episode mania. In conclusion, deficits are present in some but not all areas of cognitive functioning during the early stages of bipolar I disorder. Further research is warranted to understand the longitudinal trajectory of change from first-episode mania.

**Electronic supplementary material:**

The online version of this article (doi:10.1186/s40345-015-0024-2) contains supplementary material, which is available to authorized users.

## Review

### Introduction

The presence and impact of cognitive changes in bipolar disorder has more recently been widely appreciated. Indeed, the presence of cognitive dysfunction is particularly noteworthy given the evidence that many people with bipolar disorder start out cognitively intact or with even superior cognitive functioning (MacCabe et al. 2010). There remains an ongoing debate on the timing, pattern and significance of these changes, including whether cognitive impairments in bipolar disorder are state-dependent or trait markers of the illness.

There is substantial evidence of cognitive impairments in people who are in remission from acute episodes of bipolar disorder, supporting that euthymia may not be a period of complete recovery (Clark et al. 2002; Quraishi and Frangou 2002; Latalova et al. 2011; Malhi et al. 2007; Martinez-Aran et al. 2004; Lewandowski et al. 2011). More specifically, several meta-analyses comparing euthymic patients to healthy control (HC) participants have confirmed significant differences of medium to large effect in tasks involving processing/psychomotor speed, attention, sustained attention, verbal learning and memory, visual memory and executive functions such as: set shifting, response inhibition, verbal fluency and working memory (Arts et al. 2008; Bora et al. 2009; Mann-Wrobel et al. 2011; Bourne et al. 2013; Robinson et al. 2006; Torres et al. 2007).

These findings are in contrast to reports suggesting that asymptomatic people who subsequently will develop bipolar disorder may show minimal or no cognitive deficits prior to illness onset (MacCabe et al. 2010). Berk et al. (2007a) modified the staging model for bipolar disorder, identifying an opportunity for early intervention with the potential that early use of pharmacological treatments such as lithium carbonate may have neuroprotective properties (Swann et al. 1999; Franchini et al. 1999). However, the majority of research on cognition in bipolar disorder has been conducted on the later stages, and whilst cognitive impairments appear to worsen with illness progression (Robinson and Ferrier 2006), the extent and pattern of cognitive dysfunction in the early stages remain largely unknown.

First-episode mania (FEM) is a crucial time for the trajectory of cognitive change. Hence, identifying cognitive deficits that may be present prior to the effects of multiple episodes and prolonged exposure to psychotropic treatment is theoretically important, whilst also informing approaches to early intervention (Berk et al. 2010). To date, there has only been one meta-analysis on cognitive functioning in first-episode bipolar disorder, with the findings of deficits ranging from small to large effect for processing speed, attention, verbal learning and memory and executive functions in patients relative to HCs (Lee et al. 2014). However, this study included all phases of bipolar disorder (i.e. depression, hypomania, mania, mixed or psychosis) as a first episode, and the study was restricted to adult samples. The mean age of onset of bipolar disorder typically is in adolescence at about age 17 (Berk et al. 2007b). Perlis et al. (2004) found in their sample of 1,000 participants with bipolar disorder that approximately 40% had an early age of onset between 13 and 18 years of age, which was linked to a more severe course of illness and an increased likelihood of comorbid disorders.

This review will be the first to include adolescents with bipolar disorder and to provide a formal systematic quality assessment of the studies on cognitive impairment in FEM. The assessment of cognitive change from FEM is crucial in better understanding whether cognitive deficits are progressive or already present from the first diagnostic episode of bipolar I disorder. The main objective was to identify the degree and pattern of cognitive deficits present in FEM by systematically reviewing the literature focusing on two illness phases: cognitive functioning during the first acute manic state and in the following remission period.

### Methods

#### Search strategy

A comprehensive search of the literature on cognitive functioning in FEM was undertaken using three electronic databases, MEDLINE (Thompson Reuters Web of Knowledge), PubMed (United States Library of Medicine) and PsycINFO (Wolters Kluwer Health OvidSP). The following terms were searched in the title and abstract fields: ‘first episode mania’; ‘single manic episode’; ‘first episode bipolar disorder’; ‘early onset mania’ or ‘early onset bipolar disorder’ along with ‘neurocognition’, ‘neurocognitive’, ‘neuropsych*’, ‘cognition’, ‘cognitive’, ‘executive function’, ‘attention’, ‘memory’, ‘processing speed’, ‘intelligence’, ‘intellectual’ or ‘IQ’. The search was limited to English-language articles published between 1 January 1980 and 1 June 2014.

#### Inclusion and exclusion criteria

To be eligible for inclusion, the studies had to meet the following criteria: (1) cross-sectional study design, (2) a participant group of patients with FEM (or a first mixed episode) satisfying criteria for a diagnosis of either bipolar I disorder or schizoaffective disorder by the use of a standardised diagnostic manual, (3) a comparison sample of age and sex group-matched HCs, (4) the administration of objective and standardised cognitive tests, (5) cognitive functioning comparisons between the psychiatric group and HCs, (6) studies with more than one psychiatric group must have had made clear comparisons of the FEM patients (as a distinct group) and HCs and (7) comparison groups must have included a sample size of more than 15 participants. The exclusion criteria for FEM participants were as follows: (1) a previous medically treated manic episode and (2) a neurological disorder including severe head trauma and/or a history of epilepsy/seizures.

#### Study selection

**Figure 1:**
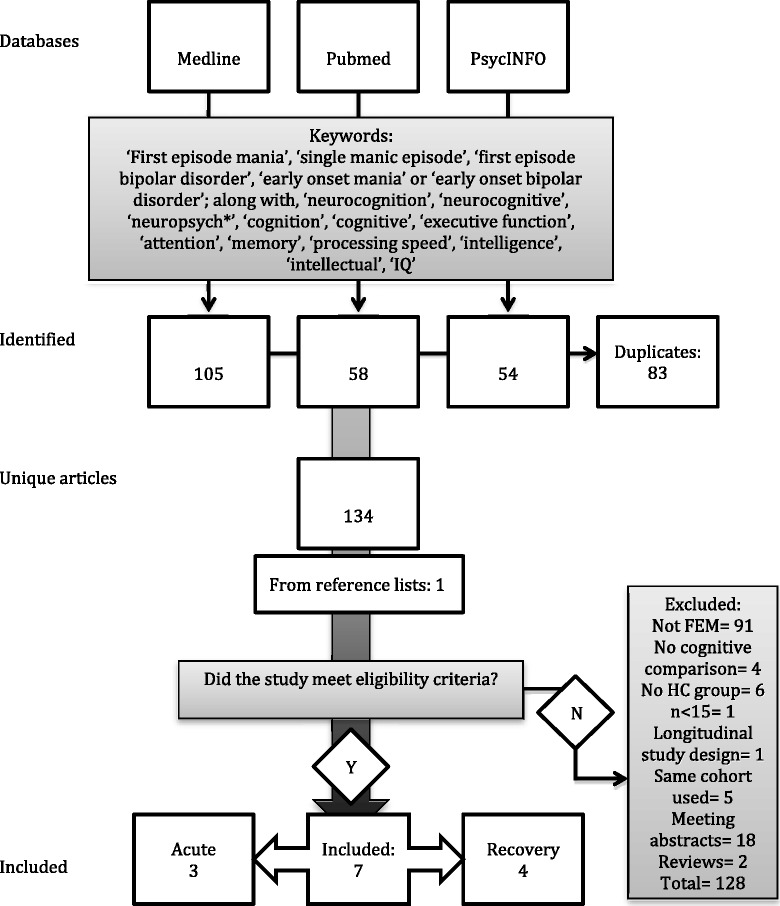
**Study selection process according to PRISMA guidelines (Moher et al.** 2009 **).**

Across all three databases, the search generated 217 journal articles that made reference to the search terms in their titles and abstracts. Of the 217 articles identified, 134 remained after duplicates were removed. Full texts of the remaining articles were retrieved and assessed to determine whether they met the inclusion and exclusion criteria for this review (see Figure 1). Reference lists of all eligible studies were checked for further relevant studies resulting in the identification of one extra article (Lopez-Jaramillo et al. 2010). The first author (RD) screened and reviewed all articles for eligibility, which were confirmed by the second author (MY). Any uncertainties or discrepancies were discussed and mutually resolved by meticulous observation of the inclusion and exclusion criteria. Studies that compared different cognitive domains, albeit of the same cohort, were deemed eligible for the review (e.g. Fleck et al. 2008; Lebowitz et al. 2001; Strakowski et al. 2008). However, when the same cohort (or a percentage of the same participants) was used across several studies that compared similar cognitive domains, the primary study with cognition as the main variable was selected for inclusion in the review to avoid repetition of results. This resulted in the exclusion of secondary studies that compared the effects of sex, traumatic events, neurosubstrates, social functioning or previous depressive episodes as variables related to cognitive functioning (Bucker et al. 2013; Bucker et al. 2014; Hellvin et al. 2013; Kozicky et al. 2013; Muralidharan et al. 2014).

#### Study participants

All eligible studies were separated into two groups: acute and remission. Participants in the acute group represented individuals experiencing an acute manic episode during the time of the cognitive assessment, whereas the participants in the remission group were predominantly euthymic at the time of testing.

#### Data extraction and analysis

A data extraction form was used to extract and record all pertinent methodological information and sample characteristics. All relevant statistical analyses from the studies (including mean, standard deviations, statistical tests, *p*-values and effect sizes) were compiled and recorded. A meta-analysis was not performed due to the restricted number of studies in the acute and remission groups and due to the large variety in cognitive tests used across the studies.

A semi validated quality assessment tool recommended by Cochrane for non-randomised studies was used to measure the quality of the included studies (Higgins and Green 2011). The Newcastle-Ottawa quality assessment scale offers a comprehensive measurement for risk of bias that can be applied to case-control and cohort study designs (Wells et al. 2006). The scale incorporates a ‘star system’ in which each study can receive up to a maximum of nine stars if all criteria have been satisfied within three categories: selection, comparability and exposure/outcome. The tool has been rated as one of the better quality assessment scales for use in systematic reviews of observational studies (Deeks et al. 2003).

### Results

#### Types of studies

In total, seven studies were considered eligible for this systematic review. Of these, three focussed on acute patients who were inpatients for FEM at the time of the cognitive testing. The four remaining studies examined participants in the period following acute FEM, though some ongoing symptomatology was present in one study.

#### Type of participants

The sample characteristics of the included studies are presented in Table 1. In total, the studies comprised 230 FEM participants and 345 HCs. The sample sizes were generally small, ranging from 16 to 50 for the FEM groups and from 16 to 110 for the HC groups. Across the studies, the average age of the HC group (from 20 to 37 years) was comparable to that of the FEM samples (from 19 to 37 years). Overall, the FEM and HC groups had a close to equal proportion of males and females (56% and 51%, respectively).

**Table 1:** **Sample characteristics of FEM patients and HCs of included studies**

**Group**	**First author (year)**	**Control participants**					**FEM participants**						
		**Total**	**Male**	**Age (years)**	**Education (years)**	**Estimated IQ**	**Total**	**Male**	**Age (years)**	**Education (years)**	**Estimated IQ score**	**YMRS score**	**Age of onset**
		***N***	***n*** **(%)**	**Mean (SD)**	**Mean (SD)**	**Score (SD)**	***N***	***n*** **(%)**	**Mean (SD)**	**Mean (SD)**	**Mean (SD)**	**Mean (SD)**	**Mean (SD)**
Acute	Fleck et al. (2008)	48	20 (41.6)	28.2 (7.9)	12.8 (1.4)	108.7 (9.0)	21	11 (52.4)	25.7 (9.2)	11.6 (2.2)	105.2 (10)	21.7 (11.8)	NR
	Lebowitz et al. (2001)	30	13 (43.3)	31.2 (8.1)	13.16 (1.1)	109.5 (8.8)	19	10 (52.6)	27.4 (7.17)	11.9 (2.0)	103.7 (8.5)	16.6 (9.1)	23.3 (6.9)
	Strakowski et al. (2008)	16	9 (56)	20 (4)	13 (3)	NR	16	12 (75)	19 (4)	11 (3)	NR	25 (7)	NR
Remission	Elshahawi et al. (2011)	50	29 (58)	NR	NR	NR	50	33 (66)	26.4 (4.7)	NR	NR	NR	NR
	Hellvin et al. (2012)	110	49 (44.5)	31.1 (9.8)	13.4 (1.9)	111.6 (4.9)	34	15 (44.12)	31.2 (9.6)	13.1 (2.2)	110.9 (6.6)	2 (0 to 28)^b^	23 (11 to 53)^b^
							21^a^	8 (38)	30.5 (10.6)	12.9 (2.3)	109.5 (5.1)	5 (0 to 19)^b^	17 (10 to 37)^b^
	Lopez-Jaramillo et al. (2010)	66	44 (66.6)	37.44 (8.57)	9.61 (3.28)	NR	24	16 (66.6)	37.04 (10.19)	10.92 (3.79)	NR	1.21 (1.5)	25.79 (9.98)
	Torres et al. (2010)	25	12 (48)	22.5 (4.8)	14.3 (2.4)	107.4 (7.7)	45	23 (51)	22.2 (3.9)	13.4 (2.4)	107.2 (7.1)	1.8 (3.7)	19.3 (4.4)

YMRS, Young Mania Rating Scale; NR, not reported. ^a^FEM participants with previously untreated manic symptoms; ^b^median (min-max).

#### Types of cognitive tests

The neuropsychological batteries comprised cognitive tests that were standardised to the general population, psychometrically sound and widely used in this patient population (Strauss et al. 2006; Mitrushina et al. 2005). The Bergen n-back test was excluded from the review due to weak construct validity (Kane et al. 2007; Jaeggi et al. 2010). The continuous visual execution task and semantic memory with associative increment test were also not included in the analysis due to the lack of information available on test description and normative data to verify that the test was standardised as per inclusion criteria. The tests used across the studies covered the following cognitive domains: processing speed, attention, learning and memory, visuospatial orientation, executive functioning and intelligence. A list of all the cognitive tests representing the aforementioned domains is available as Additional file 1: Table S1.

#### Methodological quality

The Newcastle-Ottawa criteria and total scores for each study are presented in Table 2. Overall, the methodology of the studies posed several potential biases, with at least three quality indicators omitted from each study. The mean scale score across the studies was 6 out of 9, with two studies satisfying less than half of the quality assessment markers. Details of potential methodological bias are described below.

**Table 2:** **Methodological quality assessment for the FEM studies (Newcastle-Ottawa scale)**

**Group**	**First author (year)**	**Selection**			**Comparable**		**Exposure/outcome**			**Total stars (out of 9)**
		**Validation of case definition or cohort exposure**	**Representative sample**	**Selection of controls or non-exposed cohort**	**Definition of controls or non-exposed cohort**	**Groups comparable in design and analysis**	**Assessment of exposure or outcome**	**Same method used for both groups or adequate follow-up period**	**Non-response rate or loss to follow-up**	
Acute	Fleck et al. (2008)	*	*	−	*	**	−	−	−	5
	Lebowitz et al. (2001)	*	−	−	−	**	−	*	−	4
	Strakowski et al. (2008)	−	−	*	*	**	−	*	*	6
Remission	Elshahawi et al. (2011)	−	−	−	*	**	−	*	−	4
	Hellvin et al. (2012)	*	*	*	*	**	−	*	−	7
	Lopez-Jaramillo et al. (2010)	*	*	−	*	**	−	*	−	6
	Torres et al. (2010)	−	*	*	*	**	−	*	−	6

*criteria was met; **criteria was met and awarded two stars (comparability only); −criteria was not met.

##### Selection

Two studies did not provide the details of independent validation (such as use of participant’s clinical records) to confirm the case definition (Elshahawi et al. 2011; Strakowski et al. 2008). Three of the seven studies were not broadly representative of the clinical population as they only included inpatients or patients with psychotic features (Elshahawi et al. 2011; Lebowitz et al. 2001; Strakowski et al. 2008). Lebowitz et al. (2001) omitted pertinent details of the hospital and the community from which the patients and HCs had been recruited and the period of recruitment; the definition of HCs was also not sufficient. Based on the author affiliation details provided by Lebowitz et al. (2001), it appears that the FEM participants were recruited from the same inpatient units as those from the other two acute studies. Fleck et al. (2008) did not provide clear details of the community from which the HC group were recruited. Elshahawi et al. (2011) only recruited employees from Ain Shams University Hospitals as their HC participants, whilst Lopez-Jaramillo et al. (2010) recruited the patients’ relatives as their HC group.

##### Comparability

The FEM and HC groups were well matched on several demographic variables. In addition to age and gender, the comparison groups were matched on estimated premorbid intelligence quotient (IQ) (Lebowitz et al. 2001; Torres et al. 2010; Fleck et al. 2008; Hellvin et al. 2012), education (Elshahawi et al. 2011; Hellvin et al. 2012; Lopez-Jaramillo et al. 2010; Torres et al. 2010), ethnicity (Lebowitz et al. 2001; Strakowski et al. 2008), religion (Elshahawi et al. 2011), marital status (Elshahawi et al. 2011) and occupation (Elshahawi et al. 2011; Lopez-Jaramillo et al. 2010). One study did not match groups in education (Lebowitz et al. 2001).

##### Exposure/outcome

None of the seven studies satisfied the criteria for the assessment of exposure/outcome, as the interviewer was not blind to whether the participant was a FEM patient or HC. Most studies used the same method of ascertainment for both FEM and HC participants, but this was not clearly specified in one study (Fleck et al. 2008). Furthermore, clear details regarding missing data were not provided by most studies, besides for one study (Strakowski et al. 2008).

##### Other

On the surface, the approaches used for statistical analyses seemed appropriate, and the conversion of raw scores to z-scores further standardised individual scores (Hellvin et al. 2012; Lebowitz et al. 2001; Torres et al. 2010; Lopez-Jaramillo et al. 2010). However, many of the studies failed to adjust for the effects of potential confounding variables such as premorbid IQ or clinical variables and medication effects for the FEM group. Confidence intervals and effect sizes were seldom reported. The absence of hypotheses (Elshahawi et al. 2011; Hellvin et al. 2012), *post hoc* analyses (Elshahawi et al. 2011) or the description of the type of *post hoc* analysis used (Lebowitz et al. 2001) may also be viewed as methodological weaknesses of some of the studies. Moreover, four studies comprised small sample sizes (under 30 participants) (Fleck et al. 2008; Lebowitz et al. 2001; Lopez-Jaramillo et al. 2010; Strakowski et al. 2008), and none of the studies reported statistical power to demonstrate whether their sample sizes were large enough to illustrate meaningful differences between groups.

#### Cognitive impairment in acute mania

Group differences in cognitive functioning for the acute studies are reported in Table 3. Various components of executive functioning were examined in the included studies.

**Table 3:** **Summary of findings for the FEM acute studies**

**Cognitive function**			**Controls**		**FEM**		**Statistics**		**Study**
**Domain**	**Test**	**Specific**	***n***	**Mean (SD)**	***n***	**Mean (SD)**	***p*** **value**	**Effect size**	**First author (year)**
Executive function	Wisconsin Card Sorting Test	Perseverative errors (%)	48	12.4 (10.7)	21	19.2 (12.2)	0.01^a,b^	0.59^e^	Fleck et al. (2008)
		Perseverative responses (*n*)	48	14.8 (18.9)	21	28.3 (22.6)	0.01^a,b^	0.65^e^	
		Non-perseverative errors (*n*)	48	13.1 (14.8)	21	19.5 (13.8)	0.001^a,b^	0.43^e^	
		Unique errors (*n*)	48	1.8 (5.3)	21	4.9 (8.6)	0.001^a,b^	0.47^e^	
		Failure to maintain set (*n*)	48	0.6 (0.9)	21	0.5 (1.0)	0.32^a^	0.13^e^	
	Stop signal design	Targets, correct (%)	16	50 (12)	16	44 (20)	0.13^c^	NR	Strakowski et al. (2008)
		Stops, correct (%)	16	89 (9)	16	91 (7)	0.25^c^	NR	
		Discriminability	16	0.85 (0.04)	16	0.87 (0.06)	0.27^c^	NR	
		Bias	16	0.14 (0.03)	16	0.11 (0.05)	0.17^c^	NR	
		Target, RT (ms)	16	580 (10)	16	575 (36)	0.57^c^	NR	
	Controlled Oral Word Association Test	FAS	30	39.57 (11.07)	19	38.52 (10.95)	>0.05^d^	NR	Lebowitz et al. (2001)
		Animal category	30	19.71 (3.69)	19	18.43 (3.63)	>0.05^d^	NR	

*n*, number; RT, reaction time; ms, milliseconds; NR, not reported. ^a^Kruskal-Wallis and Chi-square tests^; b^significant difference between groups after Mann-Whitney *U* test for pairwise comparisons^; c^one-sample *t*-tests; ^d^one-way or univariate analysis of variance; ^e^Cohen’s *d.*

##### Cognitive flexibility

Significant differences were identified between groups in perseverative errors, perseverative responses, non-perseverative errors and unique errors in the Wisconsin Card Sorting Test (WCST) with medium effect sizes (Cohen’s *d* ranging from 0.43 to 0.65). No significant differences were observed in the ability to maintain set.

##### Response inhibition

No significant differences were found in the number of correct target and stop signal responses, discriminability and response reaction time in the stop signal test.

##### Verbal fluency

FEM and HCs did not significantly differ in the number of correct responses and perseverative errors for both phonemic and semantic fluency on the Controlled Oral Word Association Task.

#### Cognitive impairment in remission from FEM

Cognitive functioning mean group differences between remission and HC participants are displayed in Table 4.

**Table 4:** **Summary of findings for the FEM remission studies**

**Cognitive function**			**Controls**		**FEM**		**Statistics**		**Study**
**Domain**	**Test**	**Specific**	***n***	**Mean (SD)**	***n***	**Mean (SD)**	***p*** **value**	**Effect size**	**First author (year)**
Processing speed	Colour-word interference (D-KEFS)	Colour naming	110	28.2 (4.8)	34	31.6 (7.3)	0.002^b,e^	0.08^g^	Hellvin et al. (2012)
					21^a^	32.2 (9.2)			
		Word naming	110	21.5 (3.3)	34	22.1 (3.5)	0.161^b^	0.02^g^	
					21^a^	23.1 (4.4)			
	Grooved pegboard	N/A	110	64.0 (7.7)	34	74.7 (16.0)	<0.001^b,e^	0.20^g^	
					21^a^	77.4 (16.6)			
	Stroop	Word naming	25	103.2 (13.7)	45	100.2 (12.9)	>0.05^c,f^	0.09^h^	Torres et al. (2010)
		Colour naming	25	73.7 (12.3)	45	72.5 (11.8)	>0.05^c,f^	0.07^h^	
	Trail Making Test	A	50	83.70 (4.21)	50	94.00 (7.72)	<0.001^c^	NR	Elshahawi et al. (2011)
			66	55.89 (19.99)	24	52.50 (18.27)	>0.05^d^	<0.2^i^	Lopez-Jaramillo et al. (2010)
			25	20.8 (6.4)	45	26.0 (7.8)	>0.05^c,f^	0.55^h^	Torres et al. (2010)
	WAIS/WAIS-III	Digit symbol coding	50	11.02 (2.48)	50	7.36 (0.53)	<0.001^b^	NR	Elshahawi et al. (2011)
			66	40.92 (12.92)	24	38.63 (12.37)	>0.05^d^	<0.2^i^	Lopez-Jaramillo et al. (2010)
			110	78.1 (14.6)	34	66.8 (14.8)	<0.001^b,e^	0.11^g^	Hellvin et al. (2012)
					21^a^	67.6 (14.3)			
Attention	CANTAB	RVIP	25	0.92 (0.04)	45	0.89 (0.05)	0.008^c,e,f^	0.62^h^	Torres et al. (2010)
	CVLT	Trial 1	25	7.0 (1.6)	45	6.5 (1.9)	>0.05^c,f^	0.28^h^	Torres et al. (2010)
	WAIS-III/WMS/WMS-Revised	Digit span forward	110	6.2 (1.1)	34	5.9 (1.2)	0.33^b^	0.01^g^	Hellvin et al. (2012)
					21^a^	6.0 (1.3)			
			66	5.39 (1.14)	24	5.42 (0.97)	>0.05^d^	<0.2^i^	Lopez-Jaramillo et al. (2010)
			50	6.44 (0.54)	50	4.14 (0.35)	<0.001^c^	NR	Elshahawi et al. (2011)
Learning and memory	CANTAB	Pattern recognition	25	97.0 (3.3)	45	94.6 (7.0)	>0.05^c,f^	0.27^h^	Torres et al. (2010)
		Spatial recognition	25	83.2 (11.8)	45	76.8 (15.2)	>0.05^c,f^	0.40^h^	
		Paired associates	25	4.9 (5.4)	45	9.1 (6.5)	>0.05^c,f^	0.45^h^	
	CVLT	Total trials 1 to 5	110	57.2 (9.2)	34	53.8 (13.7)	0.158^b^	0.02^g^	Hellvin et al. (2012)
					21^a^	54.0 (10.6)			
			25	58.7 (7.7)	45	51.6 (11.6)	0.004^c,e,f^	0.61^h^	Torres et al. (2010)
		Delayed recall	110	13.2 (2.4)	34	11.3 (4.2)	0.007^b,e^	0.06^g^	Hellvin et al. (2012)
					21^a^	12.5 (3.0)	>0.05^b^		
			25	12.7 (2.7)	45	10.9 (3.0)	>0.05^c,f^	0.57^h^	Torres et al. (2010)
	Rey Complex Figure	Delayed recall	109	22.8 (6.1)	34	20.8 (7.5)	0.030^b^	0.04^g^	Hellvin et al. (2012)
					21^a^	19.1 (6.0)			
		Immediate recall	66	18.33 (10.98)	24	14.79 (5.09)	>0.05^d^	<0.4^i^	Lopez-Jaramillo et al. (2010)
	WMS	Logical memory (LM)	66	10.05 (3.64)	24	8.92 (2.78)	>0.05^d^	<0.4^i^	Lopez-Jaramillo et al. (2010)
		Visual reproduction	66	8.70 (2.83)	24	7.71 (3.38)	>0.05^d^	<0.4^i^	
		Associated pairs	66	14.86 (3.46)	24	13.69 (4.15)	>0.05^d^	<0.4^i^	
		LM recognition	66	17.29 (3.13)	24	18.53 (2.14)	>0.05^d^	<0.6^i^	
	WMS-Revised	Information	50	5.98 (0.14)	50	5.32 (0.47)	<0.001^c^	NR	Elshahawi et al. (2011)
		Mental control	50	5.78 (0.76)	50	4.78 (1.27)	<0.001^c^	NR	
		Logical memory	50	13.93 (1.72)	50	11.91 (1.19)	<0.001^c^	NR	
		Visual reproduction	50	10.66 (0.96)	50	10.80 (1.01)	0.479^c^	NR	
		Total memory	50	74.51 (4.08)	50	62.93 (3.30)	<0.001^c^	NR	
	WMS-III	LM, learning	110	26.5 (6.5)	34	24.9 (7.9)	0.381^b^	0.01^g^	Hellvin et al. (2012)
					21^a^	24.9 (4.5)			
		LM, recall	110	23.8 (7.2)	34	20.9 (8.2)	0.084^b^	0.03^g^	
					21^a^	21.4 (5.3)			
Visuospatial processing	Benton	Judgement of line orientation	25	29.0 (2.0)	45	27.4 (3.0)	>0.05^c,f^	0.45^h^	Torres et al. (2010)
Executive functions	CANTAB	Stockings	25	10.4 (1.6)	45	9.0 (2.4)	0.002^c,e,f^	0.64^h^	Torres et al. (2010)
		I/E-D	25	3.1 (5.2)	45	7.8 (8.9)	0.002^c,e,f^	0.61^h^	
	Colour-word interference (D-KEFS)	Inhibition	110	49.4 (10.5)	34	59.9 (26.6)	<0.001^b,e^	0.09^g^	Hellvin et al. (2012)
					21^a^	61.1 (18.9)			
		Inhibition/switching	110	55.6 (12.5)	34	60.9 (25.4)	0.033^b^	0.04^g^	
					21^a^	65.1 (20.6)			
	Letter-number sequencing	N/A	109	11.2 (2.3)	34	9.6 (3.1)	<0.001^b,e^	0.09^g^	
					20^a^	9.4 (2.4)			
			25	12.0 (3.1)	45	10.8 (2.6)	>0.05^c,f^	0.37^h^	Torres et al. (2010)
	Spatial working memory	N/A	25	9.2 (16.7)	45	21.0 (20.1)	<0.001^c,e,f^	0.72^h^	Torres et al. (2010)
	Stroop	Conflict time	66	62.91 (13.78)	24	65.04 (11.91)	>0.05^d^	<0.2^i^	Lopez-Jaramillo et al. (2010)
		Conflict errors	66	2.02 (2.00)	24	2.25 (2.95)	>0.05^d^	<0.2^i^	
		Interference	25	49.7 (12.4)	45	47.0 (9.3)	>0.05^c,f^	0.20^h^	Torres et al. (2010)
	Trail Making Test	B	50	232.50 (23.24)	50	264.50 (40.19)	<0.001^c^	NR	Elshahawi et al. (2011)
			66	106.95 (55.88)	24	127.67 (90.48)	>0.05^d^	<0.4^i^	Lopez-Jaramillo et al. (2010)
			25	46.1 (12.6)	45	58.5 (23.8)	>0.05^c,f^	0.58^h^	Torres et al. (2010)
	WAIS-III	Digit span backward	110	4.8 (1.2)	34	4.1 (1.1)	0.009^b,e^	0.06^g^	Hellvin et al. (2012)
					21^a^	4.3 (1.2)	>0.05^b^		
	WCST	Perseveration errors	50	10.52 (3.73)	50	26.14 (6.92)	<0.001^c^	NR	Elshahawi et al. (2011)
		Perseveration responses	72	7.1 (3.7)	34	7.8 (3.5)	0.703^b^	<0.01^g^	Hellvin et al. (2012)
					15^a^	7.5 (3.3)			
		Category completion	50	5.84 (0.42)	50	4.40 (1.44)	<0.001^c^	NR	Elshahawi et al. (2011)
			72	3.7 (1.2)	34	3.8 (1.2)	0.962^b^	<0.01^g^	Hellvin et al. (2012)
					15^a^	3.7 (1.3)			
		Total errors	72	14.4 (6.4)	26	15.4 (7.2)	0.713^b^	<0.01^g^	
					15^a^	15.6 (6.2)			
	WCST (short version)	Perseverative responses	66	16.56 (6.74)	24	19.29 (8.61)	>0.05^d^	<0.4^i^	Lopez-Jaramillo et al. (2010)
		Perseverative errors	66	25.17 (7.42)	24	26.42 (8.37)	>0.05^d^	<0.2^i^	
		Categories	66	2.79 (1.21)	24	2.67 (1.24)	>0.05^d^	<0.2^i^	
	WMS/WMS-Revised	Digit span backward	66	3.08 (1.06)	24	3.83 (1.02)	0. 005^d^	0.73^i^	Lopez-Jaramillo et al. (2010)
			50	5.30 (0.79)	50	3.66 (0.48)	<0.001^c^	NR	Elshahawi et al. (2011)
	Verbal fluency (DKEFS)	Letter	110	43.0 (10.1)	34	41.3 (13.3)	0.755^b^	<0.01^g^	Hellvin et al. (2012)
					21^a^	42.3 (13.6)			
		Category	110	48.9 (8.5)	34	44.9 (11.0)	0.208^b^	0.02^g^	
					21^a^	45.8 (11.7)			
		Category switching	110	14.9 (2.4)	34	14.0 (2.8)	0.199^b^	0.02^g^	
					21^a^	14.5 (3.6)			
	Verbal fluency	Semantic	66	18.08 (3.53)	24	17.60 (3.21)	>0.05^d^	<0.2^i^	Lopez-Jaramillo et al. (2010)
		Phonological	66	12.31 (3.64)	24	11.23 (2.94)	>0.05^d^	<0.4^h^	
		FAS	25	41.4 (12.6)	45	38.5 (9.7)	>0.05^c,f^	0.23^h^	Torres et al. (2010)
Intelligence	K-BIT	Vocabulary score	25	103.5 (9.6)	45	102.3 (11.3)	>0.05^c,f^	0.09^h^	
		Matrices	25	113.4(7.7)	45	106.6(11.6)	0.01^c,e,f^	0.59^h^	
	WAIS	Verbal IQ	50	100.58 (9.71)	50	90.22 (6.02)	<0.001^b^	NR	Elshahawi et al. (2011)
		Performance IQ	50	104.06 (9.83)	50	84.86 (4.80)	<0.001^b^	NR	
		Full scale IQ	50	100.80 (8.87)	50	86.58 (4.62)	<0.001^b^	NR	
			66	99.53 (14.2)	24	96.24 (14.7)	0.002^b^	0.10^i^	Lopez-Jaramillo et al. (2010)
	WASI	Vocabulary	110	60.6 (7.3)	34	60.3 (8.8)	0.902^b^	<0.01^g^	Hellvin et al. (2012)
					21^a^	61.2 (6.6)			
		Similarities	110	38.5 (5.2)	34	37.4 (5.1)	0.555^b^	<0.01^g^	
					21^a^	37.9 (4.1)			
		Block design	110	55.6 (10.2)	34	47.2 (15.9)	<0.001^b,e^	0.11^g^	
					21^a^	46.0 (12.2)			
		Matrix reasoning	110	28.2 (3.3)	34	27.8 (5.8)	0.042^b^	0.04^g^	
					21^a^	25.7 (4.8)			
		Full scale IQ	110	111.6 (11.4)	34	108.6 (14.7)	0.159^b^	0.02^g^	
					21^a^	106.9 (9.6)			

FEM, first-episode mania; D-KEFS, Delis-Kaplan Executive System; WAIS, Wechsler Adult Intelligence Scale; CANTAB, Cambridge Neuropsychological Test Automated Battery; CVLT, California Verbal Learning Test; WMS, Wechsler Memory Scale; K-BIT, Kaufman Brief Intelligence Test; WASI, Wechsler Abbreviated Scale of Intelligence; RVIP, Rapid Information Visual Processing; I/E-D, intra/extra-dimensional test; NR, not reported. ^a^FEM participants with previously untreated manic symptoms; ^b^one-way ANOVA or univariate ANOVA; ^c^two-tailed, independent-samples *t*-test, univariate *t*-test or student *t*-test; ^d^Mann-Whitney *U* test; ^e^significant difference after Bonferroni correction, ANOVA and *t*-test; ^f^z-scores used in main analysis and effect size; ^g^eta squared; ^h^Cohen’s *d* with Hedges’ correction; ^i^effect size (ES) calculation for Mann-Whitney *U*, ES > 0.70 was considered significant.

##### Processing speed

All four studies included at least one measure of processing speed; however, the findings were mixed. Two studies identified significant differences between groups in completion time for the colour-naming task of the colour-word interference test, digit symbol coding, part A of the Trail Making Test (TMT-A) and grooved pegboard; on the contrary, two other studies found no significant difference between FEM and HC participants in colour or word naming (Stroop), digit symbol coding and TMT-A.

##### Attention

Attention span

Three of the four studies that assessed attention span found that there were no significant differences between groups in the California Verbal Learning Test (CVLT) trial I or in digit span forward. Conversely, one study reported that HCs performed significantly better than FEM patients with respect to digit span forward.

Sustained attention

A medium to large effect (Cohen’s *d* with Hedges’ correction = 0.62) was noted in rapid visual information processing, with HCs significantly surpassing the performance of FEM patients.

##### Memory

Verbal learning and memory

All four studies compared verbal learning and memory abilities in FEM patients and HCs. One study reported that patients recalled significantly less words on CVLT trials 1 to 5 compared to HCs with medium to large effect noted (Cohen’s *d* with Hedges’ correction = 0.61); though, there was no significant difference in delayed recall. On the contrary, another study reported no significant difference between groups in trials 1 to 5, though patients without a previous history of untreated manic symptoms performed significantly poorer than HCs in delayed recall, with medium effect (*η*^2^ = 0.06).

Of the three studies that used the Wechsler Memory Scale (WMS) to assess verbal memory, two reported no significant difference in WMS-III subtests. However, one study found that patients performed significantly poorer than HCs on all subtests of the WMS-Revised besides visual reproduction.

Non-verbal learning and memory

There were no significant differences between groups in spatial learning and memory as assessed by the Rey-Osterrieth Complex Figure Test in immediate and delayed recall and in the Cambridge Neuropsychological Test Automated Battery for pattern and spatial recognition and paired associates.

##### Visuospatial orientation

There was no significant difference between groups for Benton’s Judgment of Line Orientation.

##### Executive function

Cognitive flexibility

Of the three studies that measured cognitive flexibility, only one study reported a highly significant difference in WCST and in the time to complete TMT-B, with patients performing substantially worse than the HCs.

Response inhibition

Patients performed poorer than HCs in response inhibition as measured by the completion time of the colour-word interference test (*η*^2^ = 0.09). No significant difference was found between FEM and HC participants in conflict mistakes and conflict time of the Stroop interference test.

Set shifting

Patients performed significantly poorer than HCs in the attentional set shifting intra/extra-dimensional task with medium to large effect noted (Cohen’s *d* with Hedges’ correction = 0.61).

Spatial planning

Patients had significantly poorer performance on the stockings of Cambridge than HCs with medium to large effect noted (Cohen’s *d* with Hedges’ correction = 0.64).

Verbal fluency

There were no significant differences between patients and HCs on both semantic and phonological verbal fluency tasks.

Working memory (verbal and non-verbal)

FEM patients had poorer verbal working memory compared to HCs on the digit span backward task, with medium effect (*η*^2^ = 0.06) for FEM patients without a previous history of untreated manic symptoms. One study found a highly significant difference in letter-number sequencing with medium to large effect for all FEM patients (*η*^2^ = 0.09), whereas another study reported no significant difference between groups on this measure.

Spatial working memory scores were significantly poorer for the remission group compared to those for the HCs with medium to large effect (Cohen’s *d* with Hedges’ correction = 0.72).

##### Intelligence

Four studies compared the current IQ of FEM patients and HCs. One study found that there was no significant difference in verbal IQ as measured by the Kaufman Brief Intelligence Test; however, spatial reasoning (matrices) was significantly poorer for patients in remission than that for HCs with medium effect (Cohen’s *d* with Hedges’ correction = 0.59). Similarly, another study found that FEM patients and HCs performed alike on all Wechsler Abbreviated Scale of Intelligence subscales, besides block design in which HCs performed superior to patients with medium to large effect (*η*^2^ = 0.11).

On the contrary, one study reported that the FEM group performed significantly poorer on all subscales of the Wechsler Adult Intelligence Scale except for the arithmetic subtest.

#### Confounding variables

##### Clinical characteristics

There was no significant correlation between the following clinical variables and cognitive functioning: illness duration, age of illness onset, age of manic episode onset, treatment delay, time elapsed from FEM, previous depressive episode, prior hypomanic episode, mood or psychotic symptoms or substance abuse comorbidity. A potential confounding variable for cognitive flexibility in acute mania was premorbid intelligence, with a significant relationship identified for all WCST measures besides failure to maintain set.

##### Psychiatric treatment/medication

Treatment medication posed a potential confounding variable for remission patients. One study found that patients in remission taking lithium (*n* = 16) performed significantly better in spatial reasoning/orientation and executive functioning tasks than patients on divalproex (*n* = 20). No significant correlation was identified in the dose of either lithium or divalproex on cognitive performance. Patients on lithium treatment performed poorer on memory tasks compared to HCs, whilst patients treated with divalproex performed significantly poorer than HCs in executive functions, spatial reasoning and memory tasks. Patients treated with an atypical antipsychotic (*n* = 30) and those without (*n* = 15) did not differ regarding frequency of treatment and performed similarly across all cognitive domains. Whilst another study found that there were no significant differences between medicated and unmedicated FEM patients in reaction time, discriminability and bias for the response inhibition task, one study reported that increased daily dosage of antipsychotic medication was significantly correlated with a slower performance on grooved pegboard (*p* = 0.01).

### Discussion

This systematic review and quality assessment examined cognitive functioning in FEM. Based on our stringent inclusion and exclusion criteria, studies on cognitive functioning in the acute and remission phases of FEM were limited to three and four studies, respectively. All studies had limitations indexed by omitting at least three quality indicators based on the Newcastle-Ottawa scale. The cognitive assessment during the acute phase was restricted to the executive functioning domain, with the findings of impairment in cognitive flexibility but not in response inhibition and verbal fluency. Collectively, the findings were largely mixed, although individual studies during the remission phase revealed deficits in several cognitive domains. The most consistent cognitive deficit during remission was in working memory, whilst the impairments identified in sustained attention, set shifting and spatial planning were only found in one study. Another consistent finding was that verbal fluency and non-verbal memory were not impacted during remission from FEM. Due to the limited number of studies in FEM and the inconsistency of the findings during the remission phase, the widespread cognitive deficits as reported by a recent meta-analysis in first-episode bipolar disorder could not be confirmed by this review (Lee et al. 2014).

#### First acute mania and cognition

The first stage of this systematic review was to examine the impact of a first acute manic state on cognitive functioning. Acute patients with comorbid psychiatric disorders, including substance abuse within 3 months of neuropsychological testing, were excluded from the studies. Although acute and HC participants were closely matched on several demographic variables, acute patients were less educated than HCs in one study (Lebowitz et al. 2001), and no comparison was made between groups on premorbid IQ in another study (Strakowski et al. 2008). Furthermore, caution needs to be taken in the interpretation of the results due to the potential biases posed by the extant literature. Also, the severity of symptoms of the FEM patients varied substantially, with some patients presenting as floridly unwell, whilst others were under-threshold for an acute episode at the time of testing.

In this review, acute FEM patients substantially differed from HCs on all but one measure (failure to maintain set) of the cognitive flexibility task (WCST). The non-significant difference was likely attributed to a ‘flooring effect’ due to the low distribution of scores across both groups, though premorbid IQ may have had a confounding effect. Acutely manic patients and HC participants showed similar levels of impulsivity in the stop signal task. After controlling for premorbid IQ, a study by Martinez-Aran et al. (2004) revealed that individuals with chronic bipolar disorder performed worse in cognitive flexibility (WCST) and response inhibition (Stroop) during acute states of illness (mania/hypomania and depression) and in the euthymic phase when compared to HCs. However, the low inter-correlation between the stop signal and Stroop tasks indicates that the tests may be sensitive to different functions of response inhibition (Khng and Lee 2014). These findings suggest that prefrontal dysfunction associated with cognitive inflexibility, but not response inhibition, may be a trait factor of bipolar disorder that arises from the first acute episode (Soares 2003; Lyoo et al. 2004; Morice 1990).

Interestingly, even though hyperverbosity is a common feature of acute mania, a similar level of phonemic and semantic verbal fluency was reported between acute patients and HCs. The number of error intrusions and perseverative responses remained consistent between groups suggesting that FEM does not significantly impact fluency output. However, the same study revealed that acutely manic patients with multiple episodes had a significant reduction in verbal fluency relative to HCs, even when errors were included in the total number of responses. Martinez-Aran et al. (2004) identified that only depressed bipolar patients were impaired in phonemic fluency and that both depressed and euthymic patients, although not manic patients produced less semantic words than HCs.

#### Remission from FEM and cognition

In the second part of this systematic review, the extent of cognitive dysfunction in the period following acute FEM was examined. Although individual studies revealed impairments across all cognitive domains, the between study findings were inconsistent for most cognitive tasks besides working memory. For example, the impairments identified in processing speed, attention span, sustained attention, verbal immediate memory, delayed verbal memory, verbal intelligence and non-verbal intelligence were contradicted by null findings or were reported by only one study. Whilst one study found no deficit in visuospatial orientation, several studies suggested that non-verbal memory was not impacted in remission from FEM.

Regarding executive functioning, FEM patients without prior treated manic symptoms presented with a poorer working memory capacity and spatial working memory than HCs. FEM and HC participants performed similarly in response inhibition when residual symptoms were controlled, though deficits in response inhibition were identified by one study of FEM patients with ongoing symptomatology (Hellvin et al. 2012). Most studies found that there was no difference in cognitive flexibility during remission from FEM compared to HCs, besides one study of FEM patients with previous psychotic features (Elshahawi et al. 2011). Consistent with the findings in the acute phase, all studies that measured verbal fluency during remission identified that FEM patients and HCs performed similarly in both semantic and phonological categories.

In comparison, a recent meta-analysis of 12 studies on cognition in first-episode bipolar disorder in adults by Lee et al. (2014) found deficits of medium to large effect for processing speed, cognitive flexibility and attention and working memory, with smaller effects identified for deficits in verbal learning and memory and ability to maintain set and verbal fluency. Overall, there was an overlap of five out of the seven studies in the current review from the meta-analysis. It appears that the only two studies that identified impairments in processing speed and cognitive flexibility in the meta-analysis but did not met eligibility criteria for inclusion in the current review had included participants from a broader spectrum of bipolar disorders (i.e. bipolar I and II) and were not well matched to the control group in age, gender (Nehra et al. 2006), education and/or estimated verbal intelligence (Gruber et al. 2008; Nehra et al. 2006).

Similarly, the meta-analysis reported no impairment observed in visual learning and memory relative to HCs, and response inhibition was only observed in symptomatic patients (Lee et al. 2014). Although our findings identified that response inhibition was not impaired during the first acute manic episode in a sample of young people with bipolar disorder (between 15 and 35 years of age) (Strakowski et al. 2008), a deficit was observed in patients who had mainly recovered from mania but were mildly depressed at the time of testing (Hellvin et al. 2012). Interestingly, residual depressive symptoms, though not residual manic symptoms, were found to have a confounding effect on cognitive functions such as processing speed and cognitive flexibility in a meta-analysis on euthymic bipolar disorder in adults (Bourne et al. 2013).

In support of the findings on cognitive inflexibility by Elshahawi et al. (2011), a meta-analysis on bipolar I disorder by Bora et al. (2007) found that euthymic patients with previous psychotic symptoms had poorer cognitive flexibility (as measured by categories on the WCST) than euthymic patients without prior psychotic symptoms and HCs, even after controlling for confounding variables such as education, age, residual symptoms and illness severity. Fleck et al. (2008) identified that euthymic patients with bipolar disorder performed worse than HCs in the same cognitive flexibility task, although they performed better than multi-episode and first-episode acutely manic patients. Moreover, multiple-episode patients had poorer cognitive flexibility than FEM patients and HCs. Therefore, cognitive flexibility is likely to be associated with psychotic features, mood state and disease course.

Given that variations in illness severity may impact cognitive findings, there is a possibility that this was a contributing factor to the large differences between the overall study findings in this review. For example, one study reported that patients with past psychotic features, representing the more severe end of the bipolar disorder spectrum, performed significantly worse than HCs on all cognitive tests, including processing speed, memory, executive functions and intelligence (Elshahawi et al. 2011). Another study that did not report the presence of past psychotic symptoms found that the only significant difference in FEM compared to HCs was in working memory (Lopez-Jaramillo et al. 2010). A meta-analysis by Bora et al. (2010) found that patients with bipolar disorder with a history of psychosis performed poorer in processing speed, verbal memory, planning and reasoning and working memory than patients without a prior psychotic history. Another factor that may have contributed to these inconsistencies pertains to the quality of the study design. With regard to selection criteria, Lopez-Jaramillo et al. (2010) recruited healthy relatives of FEM participants as the control group, which may have influenced the findings. Past research has shown that unaffected relatives of patients with bipolar disorder may have deficits in specific cognitive tasks compared to HCs (Bora et al. 2009; Ferrier et al. 2004; Robinson and Ferrier 2006; Arts et al. 2008). The findings of cognitive deficits in relatives of patients with bipolar disorder are suggestive of pre-existing developmental or genetic vulnerability (Ferrier et al. 2004; Zalla et al. 2004). Thus, relatives are not optimal as control participants as they may have reduced the capacity to detect the extent of cognitive deficits present in the FEM group.

FEM patients and HCs were matched on several demographic variables. Hence, these variables were not likely to have influenced the cognitive findings; although, Hellvin et al. (2012) had group matched FEM patients and HCs at a ratio of 2:1. Furthermore, clinical factors such as age of onset and illness duration were not associated with poorer cognitive performance in FEM (Hellvin et al. 2012; Torres et al. 2010). This is contrary to previous reports of worse cognitive functioning associated with earlier age of onset, increased number of affective episodes, hospitalisations and duration of illness in people with bipolar disorder (Ali et al. 2001; Denicoff et al. 1999; Glahn et al. 2004; Savitz et al. 2009; Tham et al. 1997; van Gorp et al. 1998). However, differences in patients’ treatment medication were found to have an effect on cognitive functioning in FEM. Patients treated with lithium outperformed patients on divalproex on several cognitive tasks (Torres et al. 2010), whilst an increase in the daily dose of antipsychotic medication trended towards poorer processing speed in FEM patients (Hellvin et al. 2012). A study by Kravariti et al. (2005) found that a higher dose of lithium was associated with fewer errors on an executive functioning task in people with bipolar disorder, though there was no relationship between the dose of antipsychotic medication and task performance. Strakowski et al. (2008) reported no difference in response inhibition between medicated and unmedicated patients; however, the sample size was small, the treatment was not specified and the patients had only received medication for a few days prior to the cognitive assessment.

#### Cognition in FEM compared with multi-episode bipolar disorder

The findings from this review relative to meta-analyses on multi-episode bipolar disorder suggest that there may be a worsening of cognition with progression of illness (Bora et al. 2009; Mann-Wrobel et al. 2011; Arts et al. 2008; Bourne et al. 2013; Robinson et al. 2006). When comparing their findings on FEM to previously published studies on multiple-episode patients, Torres et al. (2010) reported that although premorbid/verbal IQ, attention and processing speed were similar with FEM patients, the multiple-episode patients performed worse in measures of executive functioning and verbal memory. Similarly, Hellvin et al. (2012) found that FEM and multiple-episode patients performed alike in measures of verbal recall and executive functioning, though multiple-episode patients were more impaired in verbal memory, attention and verbal fluency. Two studies in this review assessed the effects of multiple episodes on cognitive functioning in addition to FEM (Elshahawi et al. 2011; Lopez-Jaramillo et al. 2010). Their findings revealed that those with recurrent episodes performed worse in attention, processing speed and executive functions (Lopez-Jaramillo et al. 2010; Elshahawi et al. 2011) even after accounting for covariates, such as disease onset, chronicity, depression and medication (Lopez-Jaramillo et al. 2010). Moreover, a recent longitudinal study on FEM patients revealed an improvement in processing speed and executive functions relative to HCs over a 1-year period after the first acute manic episode (Torres et al. 2014). However, patients who relapsed during the 1-year follow-up period did not show improvement in cognitive functioning and those who had a longer duration of relapse for mania and hypomania showed further cognitive decline (Kozicky et al. 2014). Although there appears to be an improvement in cognitive functioning in FEM patients who have remained well, there has only been one longitudinal study on cognitive functioning in FEM. Therefore, further longitudinal studies examining cognitive functioning in FEM patients is warranted, particularly in relation to the effects of long-term medication use as well as illness course, such as relapses.

#### Limitations

Only seven studies were located by the search according to the eligibility criteria. Whist well-formulated inclusion and exclusion criteria strengthen the aims and overall findings, they also limit the ability to confer associations between studies on cognitive functioning in juvenile bipolar disorder, first-episode affective disorders and in first-episode bipolar disorders when episode polarity was not specified. Another limitation relates to the cross-sectional nature of the studies, meaning that there was no assessment of cognitive functioning prior to the onset of the illness. It is unclear from this review whether the cognitive deficits in the early stages of bipolar disorder had commenced after illness onset or were present during the prodromal or premorbid phase. Additionally, due to the shared variance between cognitive functions such as attention and processing speed (Antila et al. 2011), there can be a loss of specificity in relation to the exact area of cognition impacted.

## Conclusions

This systematic review has revealed a relatively robust deficit in working memory, with evidence of impairment in several other cognitive domains in some, but not all studies included in this review. There was no evidence of dysfunction in verbal fluency during both the acute state and remission period of a FEM, and non-verbal memory does not appear impacted during remission. This suggests a finite window for potentially neuroprotective effects as past literature on chronic bipolar disorder has identified deficits in both these domains, highlighting the theoretical importance of early intervention and treatment adherence. Longitudinal research on cognitive functioning after the onset of bipolar I disorder is needed in order to assess the extent to which cognitive deficits progress over time.

## Additional file

Additional file 1: Table S1. Cognitive domains and functions represented by the neuropsychological test utilised in the studies.

## Abbreviations

CVLT: California Verbal Learning Test
FEM: first-episode mania
HC: healthy control
IQ: intelligence quotient
TMT: Trail Making Test
WCST: Wisconsin Card Sorting Test
WMS: Wechsler Memory Scale
