# Supplementary material for: Cognitive impairment in first-episode mania: a systematic review of the evidence in the acute and remission phases of the illness
Source: Int J Bipolar Disord. 2015 Apr 25;3:9. doi: 10.1186/s40345-015-0024-2 (PMC4408302; doi:10.1186/s40345-015-0024-2)
Supplement: Additional file 1: Table S1. — Cognitive domains and functions represented by the neuropsychological test utilised in the studies. [file 40345_2015_24_MOESM1_ESM.docx]

Supplementary Table. Cognitive domains and functions represented by the neuropsychological test utilised in the studies

| Cognitive domain | Cognitive function | Neuropsychological test utilised | First author (year) |
| --- | --- | --- | --- |
| Intelligence | Full scale IQ | Wechsler Adult Intelligence Scale (WAIS) | Elshahawi (2011)^31^; Lopez-Jaramillo (2010)^17^ |
|  |  | Wechsler Abbreviated Scale of Intelligence | Hellvin (2012)^30^ |
|  | Verbal IQ | Kaufman Brief Intelligence Test, vocabulary | Torres (2010)^32^ |
| Speed of processing information | Processing speed | Trail Making Test- A | Elshahawi (2011)^31^; Lopez-Jaramillo (2010)^17^; Torres (2010)^32^ |
|  |  | Digit symbol coding (WAIS/WAIS-III) | Elshahawi (2011)^31^; Hellvin (2012)^30^; Lopez-Jaramillo (2010)^17^ |
|  |  | Grooved Pegboard | Hellvin (2012)^30^ |
|  |  | Colour-Word Interference Test (from D-KEFS), colour naming and word reading | Hellvin (2012)^30^ |
|  |  | Stroop, colour/word naming trial, number correct | Torres (2010)^32^ |
| Attention | Attention span | California Verbal Learning Test-second edition (CVLT-II), trial I words recalled | Torres (2010)^32^ |
|  |  | Digits forward subtest (from WMS/WMS-R) | Elshahawi (2011)^31^; Lopez-Jaramillo (2010)^17^ |
|  |  | Digit Span forward subtest (from WAIS) | Hellvin (2012)^30^ |
|  | Sustained attention | CANTAB rapid visual information processing | Torres (2010)^31^ |
| Learning and memory | Verbal learning and recall memory | CVLT-II recall trials 1-5 and delayed free recall | Hellvin (2012)^30^; Torres (2010)^17^ |
|  |  | Wechsler Memory Scale (WMS); WMS-third edition; WMS-Revised (WMS-R) | Elshahawi (2011)^31^; Lopez-Jaramillo (2010)^17^ |
|  |  | Visual reproduction subtest (from WMS) | Lopez-Jaramillo (2010)^17^ |
|  |  | Logical memory (from WMS/WMS-III), verbal learning and recall | Hellvin (2012)^30^; Lopez-Jaramillo (2010)^17^ |
|  | Nonverbal learning, memory | CANTAB Spatial and pattern recognition memory, and paired associates learning | Torres (2010)^31^ |
|  |  | Rey-Osterrieth Complex Figure Test | Hellvin (2012)^30^; Lopez-Jaramillo (2010)^17^ |
| Visual perception | Visuospatial orientation | Benton judgment of line orientation | Torres (2010)^31^ |
| Executive function | Verbal fluency | Controlled Oral Word Association Test | Lebowitz (2001)^29^ |
|  |  | FAS verbal fluency test | Torres (2010)^32^ |
|  |  | Verbal fluency (from D-KEFS) | Hellvin (2012)^30^ |
|  |  | Semantic and Phonological verbal fluency test | Lopez-Jaramillo (2010)^17^ |
|  | Working memory | Digits backward subtest (from WMS) | Lopez-Jaramillo (2010)^17^ |
|  |  | Digit Span Backward subtest (from WAIS) | Hellvin (2012)^30^ |
|  |  | Letter-Number sequencing | Hellvin (2012)^30^; Torres (2010)^32^ |
|  |  | CANTAB spatial working memory | Torres (2010)^32^ |
|  | Planning ability | CANTAB stockings of Cambridge | Torres (2010)^32^ |
|  | Cognitive flexibility/attentional set shifting/response inhibition | Trail Making Test- B | Elshahawi (2011)^31^; Lopez-Jaramillo (2010)^17^; Torres (2010)^32^ |
|  |  | Wisconsin Card Sorting Test | Elshahawi (2011)^31^; Fleck (2008)^27^; Hellvin (2012)^30^; Lopez-Jaramillo (2010)^17^ |
|  |  | CANTAB intra-/extra-dimensional | Torres (2010)^32^ |
|  |  | Stroop interference, number correct | Torres (2010)^32^ |
|  |  | Stroop interference, mistakes | Lopez-Jaramillo (2010)^17^ |
|  |  | Colour-Word Interference Test, inhibition and inhibition/switching subtests (from D-KEFS) | Hellvin (2012)^30^ |
|  |  | Stop-signal test | Strakowski (2008)^28^ |

DKEFS= Delis Kaplan Executive Functioning System; CANTAB= Cambridge Neuropsychological Test Automated Battery
